# Environmental sustainability of European production and consumption assessed against planetary boundaries

**DOI:** 10.1016/j.jenvman.2020.110686

**Published:** 2020-09-01

**Authors:** Serenella Sala, Eleonora Crenna, Michela Secchi, Esther Sanyé-Mengual

**Affiliations:** European Commission, Joint Research Centre (JRC), Via E. Fermi 2749, 21027, Ispra, Italy

**Keywords:** Consumption patterns, Impact assessment, Life cycle assessment based indicator, Sustainable development goals, Absolute sustainability, Carrying capacity

## Abstract

The planetary boundaries (PBs) represent a well-known concept, which helps identify whether production and consumption systems are environmentally sustainable in absolute terms, namely compared to the Earth's ecological limits and carrying capacity. In this study, the impacts of production and consumption of the European Union in 2010 were assessed by means of life cycle assessment (LCA)-based indicators and compared with the PBs. Five different perspectives were adopted for assessing the impacts: a production perspective (EU Domestic Footprint) and four distinct consumption perspectives, resulting from alternative modelling approaches including both top-down (input-output LCA) and bottom-up (process-based LCA). Life cycle impact assessment (LCIA) results were assessed against LCIA-based PBs, which adapted the PBs framework to the LCIA indicators and metrics of the Environmental Footprint method (EF). Global environmental impacts transgressed several LCIA-based PBs. When assessing the overall environmental impacts of EU consumption compared to the global LCIA-based PBs, impacts of EU consumption related to climate change, particulate matter, land use and mineral resources were close or already transgressed the global boundaries. The EU, with less than 10% of the world population, was close to transgress the global ecological limits. Moreover, when downscaling the global PBs and comparing the impacts per capita for an average EU citizen and a global one, the LCIA-PBs were significantly transgressed in many impact categories. The results are affected by uncertainty mainly due to: (a) the intrinsic uncertainties of the different LCA modelling approaches and indicators; (b) the uncertainties in estimating LCIA-based PBs, due to the difficulties in identifying limits for the Earth's processes and referring them to LCIA metrics. The results may anyway be used to define benchmarks and policy targets to ensure that consumption and production in Europe remains within safe ecological boundaries, as well as to understand the magnitude of the effort needed to reduce the impacts.

## Highlights

• Planetary Boundaries help quantify the environmental sustainability of consumption.
• We developed LCIA-based planetary boundaries for evaluating the EU consumption.
• EU consumption occupies a high share of the safe operating space globally available.
• Planetary boundaries are fundamental to support policy making towards sustainability.
• LCA-based planetary boundaries show intrinsic uncertainties.

## Graphical abstract

**Figure:**
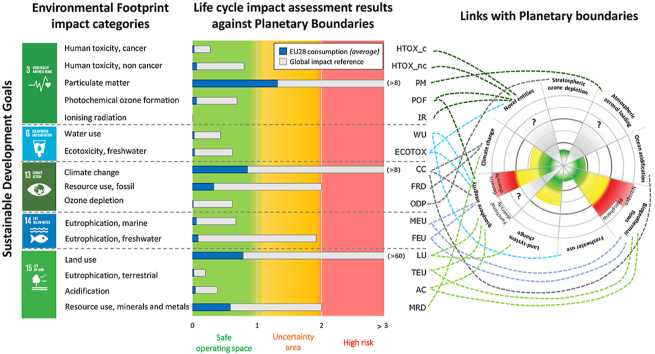

## 1 Introduction

The sustainability of production and consumption patterns is a central topic both in the scientific literature and the policy debate. As social and economic development is increasingly exerting pressure on the environment, thus leading to relevant and sometimes irreversible changes, a transition towards a more responsible consumption and production is needed, as urged by the United Nations (UN) Sustainable Development Goal (SDG) 12 (UN, 2015). This transition requires a systematic approach (EEA, 2017) to assess carefully to what extent the consumption patterns are environmentally sustainable. A systematic and holistic method as life cycle assessment (LCA) (ISO, 2006a, 2006b) is considered pivotal to address the sustainability of production and consumption in a comprehensive manner (Sala et al., 2013a; 2013b) and to estimate the environmental footprint addressing both pressures and impacts. LCA allows quantifying the potential environmental impacts, as consequences of human-driven environmental pressures, along the entire life cycle of products or services, thus including the impacts occurring along the supply chains in multiple territories (Bjørn et al., 2015). At the EU level, LCA is recommended as a tool for policy impact assessment in the Better Regulation (European Commission Ec, 2015) and the Environmental Footprint (EF) method has been developed as reference (EC, 2017) to assess environmental performance of products and organisation. However, notwithstanding LCA may support the evaluation of the potential impacts of a product or a system, and a relative comparison in terms of eco-efficiency thereof, this is not enough to define how sustainable they are beyond relative terms (Hauschild, 2015).

This requires the identification of absolute sustainability references against which assessing the impacts. Recently, it has been proposed to combine the planetary boundaries (PBs) framework with LCA, towards the so-called “absolute sustainability assessment” (Bjørn et al., 2015, Ryberg, 2018), as well as to define which path to follow for remaining within these boundaries.

Therefore, this paper focuses on and discusses the role of PBs within LCA. The aim of this study is twofold, namely: i) to compare alternative LCA-based options (herein referred as indicators) to assess the impacts and the absolute environmental sustainability of the EU-28 production and consumption system; and ii) to present a complete list of life cycle impact assessment (LCIA)-based PBs in relation to the 16 LCIA impact categories adopted in the EF method. Current limitations and future research needs are also illustrated.

### 1.1 The planetary boundaries framework

One of the main challenges in assessing the sustainability of consumption is the definition of a reference point, which enables the quantification of the environmental performance of the production and consumption system with respect to the Earth carrying capacity, in absolute terms. The PBs, firstly developed by Rockström et al. (2009), and updated by Steffen et al. (2015), represent a well-known concept, which supports such approach. By defining the “safe operating space” for human development, based on the planet's bio-physical processes (Rockström et al., 2009), it provides a science-based reference of the risks that human interventions will substantially alter the Earth's system (Steffen et al., 2015). The PBs framework thus considers nine among the Earth system processes, each of them embracing one or several SDGs (UN, 2015). Each Earth's process is associated with a defined ecological limit at global or regional level, set according to the precautionary principle and allowing for acceptable societal development. Limits are measured through control variables, namely metrics which quantify the state, pressure or driving forces of the environment depending on the Earth’ processes (Dao et al., 2018). Beyond the boundaries proposed by Steffen et al. (2015), other estimates have been developed, focusing on specific issues and addressing countries' performance (e.g. the phoshorous exceedance footprint of Li et al. (2019) which measures countries' contributions to the transgression of the PB for phosphorus; and the degree of reaching SGDs 6 on clean water and sanitation in a country where the PB for water has been trangressed, Roy and Pramanick (2019).

Some authors criticize the PBs framework by pointing out possible limitations of the overall approach, e.g. the lack of common metrics that could be applied consistently across different spatial scales, the need of understanding and accounting for the interactions between PBs (see e.g. Clift et al., 2017; Mace et al., 2014; Montoya et al., 2018; Pickering and Persson, 2019). However, there is an important added value in the message behind the ecological concept that the PBs community is reinforcing, namely the existence of tipping points and critical capacities of the Earth to sustain human production and consumption patterns. Considering these tipping points is necessary when defining aspirational targets in environmental protection and biodiversity conservation policy. This would allow addressing critical processes sustaining life on Earth in policy-making.

The PBs concept and the possibility of defining the absolute environmental sustainability of the consumption systems have gained growing interest in the scientific and grey literature. It is acknowledged that methods to implement the PBs theory in sustainability assessment are still an open challenge (Li et al., 2019). To overcome such limitations, new initiatives to use the PBs have been developed to contribute to the current discussion. For instance, Springmann et al. (2018) assessed the sustainability of global food consumption, analysing several options for reducing the environmental effects of the food system while keeping them within the safe operating space of the PBs. Dao et al. (2018) assessed the absolute environmental sustainability of Swiss consumption with respect to the PBs in a context of strategic planning.

The PBs are also cardinal in the achievement of the goals foreseen e.g. in the European 7th Environment Action Programme (7th EAP) (EU, 2013) (i.e. leading to a Europe where citizens may “live well within the limits of the planet”). However, besides studies at country scale (e.g. Nykvist et al., 2013; Lucas and Wilting, 2018), only one study was found dealing with the environmental impacts and the transgression of PBs at the level of the entire European Union. At EU scale, Hoff et al. (2014) collected literature data on environmental pressures and impacts of the EU to perform a partial comparison with global limits by employing the PBs as initial framework. Global PBs were distributed equally as PB per capita and global averages were employed when no PB was available for a specific impact (e.g. materials use). Consumption-based impacts were only available for climate change, water use and land use. In general, EU countries mainly exceeded global limits. However, no study has comprehensively addressed the assessment of the environmental impacts of EU consumption from a PBs perspective yet.

### 1.2 Using the planetary boundaries in life cycle assessment

**Fig. 1:**
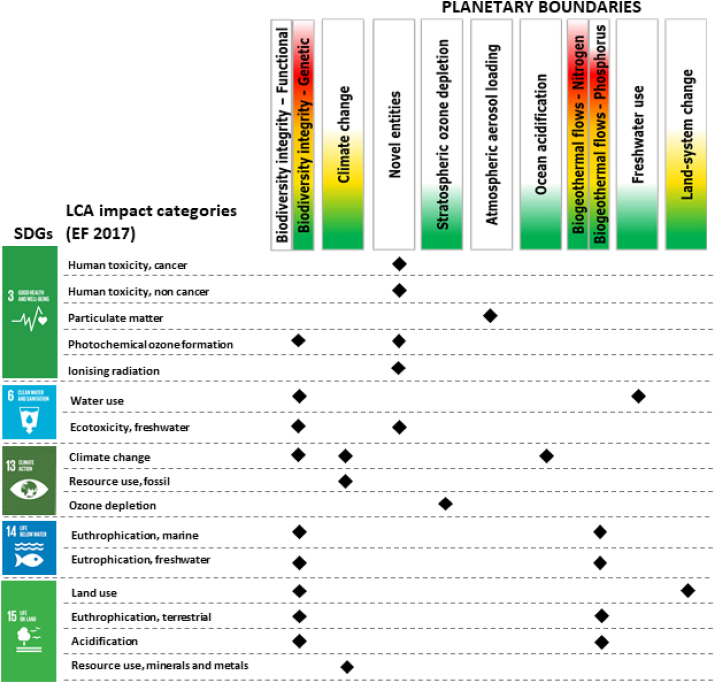
Connection between the LCIA impact categories of the Environmental Footprint method (EF), the Sustainable Development Goals (SDGs), and the planetary boundaries (PBs).

Connecting LCA impact categories, namely the categories through which environmental impacts of products and services are assessed (e.g. climate change, resource use), SDGs, and PBs helps answer the question whether EU consumption is environmentally sustainable not only in relative but also in absolute terms. Fig. 1 details the relation and interlinkages between SDGs, PBs, and LCA, by means of the link with the EF method impact categories. The nine PBs showed relation with the 16 EF impact categories, which are then related to specific SDGs addressing human health and ecosystems quality. With regard to ocean acidification, the corresponding PB was linked to climate change due to their strong cause-effect relationship. There was no link with the impact category on acidification as the underpinning LCIA model only covers terrestrial acidification.

Employing the PBs as reference for assessing LCA results can be done in two different ways.

On the one hand, the ecological limits of PBs can be translated into LCIA metrics to develop LCIA-based PBs, coherently with the impact assessment modelling underpinning each category. Following this rationale, Bjørn and Hauschild (2015) and Vargas-Gonzalez et al. (2019) adapted different impact categories based on guardrails from the policy context (e.g. EEA, 1998; WHO, 2006) and the existing literature (e.g. Rockström et al., 2009), uncovering several limitations. Bjørn and Hauschild (2015) defined absolute sustainability thresholds by identifying and quantifying for some impact categories the carrying capacity, defined as “the maximum sustained environmental intervention a natural system can withstand without experiencing negative changes in structure or functioning that are difficult or impossible to revert” (p. 1005).

On the other hand, LCIA metrics can be adapted to the ecological limits of PBs by developing specific impact assessment models. Ryberg et al. (2018a) developed a LCIA method linked to the PBs framework (PB-LCIA) by calculating characterization factors to estimate the environmental impacts in terms of the nine PBs.

Examples of applications of PBs to LCA exist, in which some impact categories have been addressed. For instance, Wolff et al. (2017) evaluated the absolute environmental sustainability of a food retail company located in France, putting the LCA results for 16 impact categories in perspective of the PBs. Ryberg et al. (2018b) applied the PB-LCIA method to an industry case study, highlighting the relevance of allocation methods for downscaling PBs. These efforts have been applied to different existing LCIA methods. However, no studies have adapted yet the PBs framework to the Environmental Footprint LCIA method.

In this study, we assessed the transgression of the PBs by the environmental impats of EU production and consumption. The calculation required the development of a set of LCIA-based PBs adapted to the models and the indicators of the EF LCIA method (EF reference package 2.0) (EC, 2013; EC, 2017; Fazio et al., 2018; Sala et al., 2019b). The EF is an LCA-based method for quantifying the environmental impacts of products, goods or services, by addressing 16 impact categories at the midpoint level such as climate change, acidification, ecotoxicity etc.

## 2 Methodology

This section describes the three dimensions of assessing the PBs - as detailed in Häyhä et al. (2016) - that have been considered: the socio-economic (i.e. modelling of environmental burdens of a given production and consumption system), the biophysical (i.e. estimations of the PBs), and ethical (i.e. allocation of the PBs to lower scales).

The environmental impacts of EU-28 consumption in 2010 were assessed by means of different LCA-based indicators developed by the European Commission Joint Research Centre (EC-JRC), as briefly presented in Section 2.1.

Then, the environmental sustainability of the EU-consumption was evaluated from both relative and absolute environmental sustainability perspectives: firstly, by comparing the LCIA indicators’ results with the respective global impact references (section 2.1.4); and, secondly, by contrasting them with the LCIA-based PBs, extensively presented in Section 2.2.

Finally, the allocation principle adopted is specified in Section 2.3.

### 2.1 Socio-economic dimension: life cycle indicators assessing the impacts of EU production and consumption

A set of LCA-based indicators was developed to monitor the progress towards decoupling economic growth from resource use and associated environmental impacts of EU production and consumption (Sala et al., 2019a; Sanyé-Mengual et al., 2019). The set of indicators included both territorial and consumption perspectives, employing either process-based LCA or input-output-based LCA and covering either final consumption or household consumption only. Table 1 summarizes the differences within the five indicators considered in this study.

**Table 1:** Life cycle indicators and their main features, used for estimating the environmental impacts of the EU consumption system.

Life cycle-based indicator	Modelling approach	Perspective	Scale & focus of the assessment	Source of data for the estimation	Reference year	EF impact category coverage	Limits of the estimation
Domestic Footprint	Bottom-up	Territorial	Country	Statistical data, models for emission estimation	2010	16	Emissions and resource extraction are taken into account within the boundary of a country
Consumer Footprint	Bottom-up	Consumption-based (products)	Products	Representative products and five areas of consumption (i.e. food, mobility, housing, household goods, appliances)	2010	16	The selection is restricted to representative products, potentially leading to incomplete estimation of the overall environmental impacts
Consumption Footprint bottom-up	Bottom-up	Combination of territorial and consumption-based (products)	Apparent consumption	Territorial for domestic, and product-based for trade	2010	16	Potential high uncertainties deriving from merging the (domestic) statistically based inventoy with the LCA inventory. Existing discrepancy in the coverage of emissions and resources. Limited number of products that can be modelled.
Consumption Footprint top-down	Top-down				2010 (domestic), 2011 (trade)	14 (ozone depletion and ionising radiation are excluded)	
Final consumption I/O Footprint	Top-down	Consumption-based (sectors)	Sectors	Based on environmentally-extended I/O tables	2011^a^	14 (ozone depletion and ionising radiation are excluded)	The sector- based approach is usually associated with a relatively limited coverage of emissions and resource.

a Due to the lowest uncertainties in the estimation of impacts, with respect to other years.

A life cycle-based approach entails the specification of the goal and scope of the study, as well as selection of an approach to build the inventory (pressures in terms of emissions and resources use associated to the consumption) and to assess the impacts (models adopted to assess the extent to which the pressure may generate a potential impact). The methodological aspects of each LCA phase are detailed in the following sections.

#### 2.1.1 Goal and scope

The goal and scope of the study was to assess the environmental impacts of the European Union consumption in 2010, including the comparison with global impacts and with LCIA-based PBs.

#### 2.1.2 Life cycle inventory

Regarding the inventory, several modelling approaches have been adopted to estimate the pressure, namely the emissions to air, water and soil, and the resources associated to EU consumption. The modelling of environmental pressures built on either bottom-up or top-down modelling approaches, based on different perspectives, scales and data sources (Table 1).

**Fig. 2:**
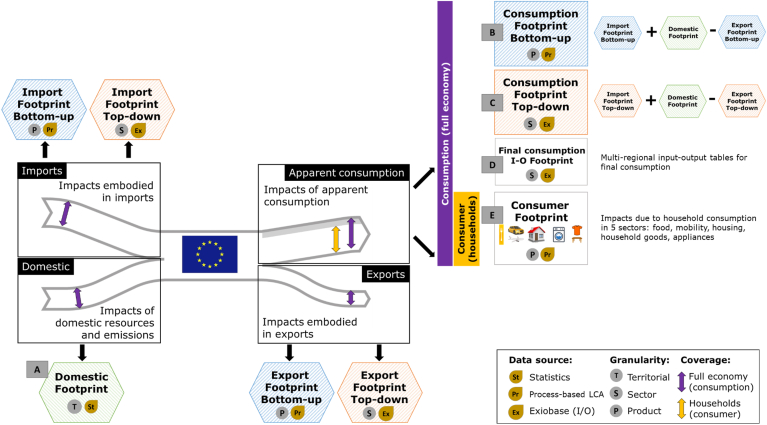
Scheme of the five life cycle-based indicators: A) Domestic Footprint, B) Consumption Footprint bottom-up, C) Consumption Footprint top-down, D) Final consumption I/O Footprint, and E) Consumer Footprint. Data source, granularity and coverage are detailed for each indicator and consumption element. Based on Sala et al. (2019a).

Fig. 2 details the data sources, granularity and coverage of the five indicators considered in this assessment:

A) Domestic Footprint: compiling statistical data of environmental pressures and resources use collected for the entire EU territory (production perspective);
B) Consumption Footprint bottom-up: combining the production perspective (domestic impacts) with product-based estimates of imports and exports (Corrado et al., 2019), namely adding the impacts due to imported goods to the domestic estimates and subtracting that of exported ones;
C) Consumption Footprint top-down: combining the production perspective (domestic impacts) with environmentally extended multi-regional input-output (EE-MRIO)-based estimates of impact and exports, namely adding the impacts due to imported goods to the domestic estimates and subtracting that of exported ones (Beylot et al., 2019);
D) Final consumption I/O Footprint: allocating emissions and resources to economic sectors, such as in the EEIO tables, with a top-down approach (input-output LCA-based);
E) Consumer Footprint: accounting for the impacts of consumption by means of the LCA of representative products in different areas of consumption, namely a basket of products approach (see Sala and Castellani (2019)), with a fully bottom-up approach (process-based LCA).

Details on the methodological differences and the implications for the choice of the methodology are reported in Sala et al. (2019a), Beylot et al. (2019), and Castellani et al. (2019). The selected indicators represent the following methodological aspects:

- Data collection considering the system boundaries with a production-based perspective (i.e., considering the domestic environmental pressures and resources use taking place within the territorial borders) or a consumption-based one (i.e., including the trade flows)
- Consumption-based approaches, i.e. where the human-driven impacts generated along the life cycle of a product or service are allocated to the final consumer (namely, an average European citizen), can focus on the consumption taking place at households (i.e., household consumption) or involve the entire consumption (i.e., final consumption including households, government and non-governmental organizations)
- LCI data for the trade components can be collected following two approaches:
  o A bottom-up approach considers process-based LCA, where the life cycle of individual products was evaluated and up-scaled to represent consumption according to statistics
  o A top-down approach estimates the environmental flows of trade by employing MRIO-based LCA

#### 2.1.3 Life cycle impact assessment

Regarding the estimate of the potential environmental impacts, the LCIA models as in the EF method (EF reference package 2.0) (EC, 2017; Fazio et al., 2018; Sala et al., 2019b) were adopted. The EF method covers 16 environmental impact categories, namely: climate change; stratospheric ozone depletion; particulate matter; ionising radiation, human health effects; photochemical ozone formation; acidification; eutrophication (terrestrial, freshwater, marine); ecotoxicity, freshwater; human toxicity (cancer and non-cancer effects); land use; water use; and resource use (minerals and metals, fossils). The same impact assessment model and metrics of the EF 2.0 were implemented for the different impact categories, apart from land use (detailed in section 2.2.2). For the land use category, the same underpinning model of the EF 3.0 was applied, namely the updated version of the LANCA model (Horn and Maier, 2018) as in De Laurentiis et al. (2019). However, as explained hereinafter only the soil erosion impact was quantified.

For the LCA-based indicators involving MRIO data (i.e. Consumption Footprint top-down and Final consumption I/O Footprint), ozone depletion and ionising radiation categories were excluded due to the lack of environmental data regarding the elementary flows underpinning these impact categories in Exiobase v3.0 (Merciai and Schmidt, 2018). Therefore, only 14 indicators of the EF method were considered (Table 1).

#### 2.1.4 Comparison of impact against global impacts

The relevance of the impact generated by European consumption could be assessed from a global perspective. In this study, the environmental impacts of EU consumption were compared with global environmental impacts as calculated by Sala et al. (2017) and Crenna et al. (2019) (see supplementary material SM1 for further details). The global environmental impact for the land use category was recalculated, as detailed in section 2.2.2.

### 2.2 Biophysical dimension: planetary boundaries and specific assumptions

A comprehensive framework for PBs is available in the current literature (Steffen et al., 2015). Table 2 details the nine Earth's processes under consideration in the PBs in terms of control variable, unit, PB limits, PB uncertainty zone and nature of limit. The current value of the control variable and whether the PB has been integrated already in LCA literature are also indicated.

**Table 2:** Overview of the planetary boundaries (PBs), as developed by Rockström et al. (2009) and updated by Steffen et al. (2015).

Earth's process	Control variable	Unit	PB limits	PB uncertainty zone	Nature of limit	Current value	Status compared to the limit	Integration in LCA in literature
Climate change	Atmospheric carbon dioxide (CO_2_) concentration	ppm CO_2_	350	350–450	Upper	398.5	Uncertainty zone	Yes
	Change in radiative forcing	W/m^2^	1	1–1.5	Upper	2.3	High risk	Yes
Change in biosphere integrity	Genetic diversity: extinction rate	E/MSY (extinctions per million species-year)	10	10–100	Upper	100–1000	High risk	No
	Functional diversity: Biodiversity intactness index	%	90	90–30	Lower	84	Uncertainty zone	No
Stratospheric ozone depletion	Stratospheric ozone (O_3_) concentration	DU (Dobson unit)	275.5	275.5–261	Lower	283	Safe operating space	Yes
Ocean acidification	Carbonate ion concentration, average global surface ocean saturation state with respect to aragonite	% of the pre-industrial aragonite saturation state	80	80–70	Lower	84	Safe operating space	No
Biogeochemical flows (N and P cycles)	Nitrogen (N) global: industrial and intentional biological fixation of N	Tg N/year	62	62–82	Upper	150	High risk	Yes
	Phosphorus (P) global: P flow from freshwater systems into the ocean	Tg P/year	11	11–100	Upper	22	Uncertainty zone	Yes
	Phosphorus (P) regional: P flow from fertilizers to erodible soil	Tg P/year	6.2	6.2–11.2	Upper	14	High risk	Yes
Land-system change	Global: area of forested land as % of original forest cover	%	75	75–54	Lower	62	Uncertainty zone	Yes
	Biome: area of forested land as % potential forest	%	50	50–30	Lower	–		–
Freshwater use	Global: maximum amount of consumptive blue water use	km^3^/year	4000	4000–6000	Upper	2600	Safe operating space	Yes
	Basin: blue water withdrawal as % of mean monthly river flows	%	30	30–60	Upper	–		–
Atmospheric aerosol loading	Global: Aerosol Optical Depth (AOD)	AOD	–	–	–	–		–
	Regional: AOD as a seasonal average over a region	AOD	0.25	0.25–0.50	Upper	0.30	Uncertainty zone	–
Introduction of novel entities	Not defined yet	–	–	–	–	–		–

The PBs with metrics linked to ecological limits cannot be used directly for assessing the sustainability of EU consumption from a life cycle perspective, i.e. comparing the LCIA results against the PBs. This is related to two main aspects: first, there is a need to map the different coverage of Earth's processes of the defined PBs and the LCIA models; and second, a link between the different metrics and indicators used for the PBs and the models adopted for LCIA is required. Identifying the link and adapting both indicators and metrics were performed in this study to be compliant with EF requirements and consistently with the impact assessment models underpinning the EF impact categories (i.e. EF reference package 2.0).

LCIA-based PBs were developed for all the 16 impact categories of the EF method (Table 3). Five of them (i.e. freshwater ecotoxicity, climate change, ozone depletion, marine and freshwater eutrophication) were taken from the available literature (Bj*ø*rn and Hauschild, 2015); while the remaining were recalculated or adapted to the EF impact assessment framework, as detailed in the following sections. The set of PBs reported in Table 3 entails both those for which there is a matching between the EF impact categories and the PB defined by Rockström et al. (2009) and those reported in other studies defining carrying capacities. The references underpinning the PBs are detailed in Table 3.

**Table 3:** Planetary boundaries (PBs) adapted to the EF metrics of each impact category, available for comparing LCIA results. The order in which the PBs are presented herein is in accordance with Table 2.

EF impact category	Abbreviation	Unit	Indicator^b^	PB	PB per capita^a^	Sources	Underpinning reference used in the sources	PB classification
Climate change	CC	kg CO_2_ eq	Radiative forcing as Global Warming Potential (GWP100)	6.81E+12	9.85E+02	Bjørn and Hauschild (2015)	Rockström et al. (2009)	Climate action, water and terrestrial life protection
Ozone depletion	ODP	kg CFC-11 eq	Ozone Depletion Potential (ODP)	5.39E+08	7.80E-02	Bjørn and Hauschild (2015)	Rockström et al. (2009)	Climate action, water and terrestrial life protection
Eutrophication, marine	MEU	kg N eq	Fraction of nutrients reaching marine end compartment (N)	2.01E+11	2.90E+01	Bjørn and Hauschild (2015)	De Vries et al. (2013)	Climate action, water and terrestrial life protection
Eutrophication, freshwater	FEU	kg P eq	Fraction of nutrients reaching freshwater end compartment (P)	5.81E+09	8.40E-01	Bjørn and Hauschild (2015)	Struijs et al. (2011)	Climate action, water and terrestrial life protection
Eutrophication, terrestrial	TEU	molc N eq	Accumulated Exceedance (AE)	6.13E+12	8.87E+02	recalculated by Bjørn (2017)	Bouwman et al. (2002)	Cclimate action, water and terrestrial life protection
Acidification	AC	molc H^+^ eq	Accumulated Exceedance (AE)	1.00E+12	1.45E+02	recalculated by Bjørn (2017)	Bouwman et al. (2002)	Climate action, water and terrestrial life protection
Land use	LU	kg soil loss	Soil erosion	1.27E+13	1.84E+03	Bjørn and Hauschild (2015)	Verheijen et al. (2009)	Climate action, water and terrestrial life protection
Water use	WU	m^3^ world eq	User deprivation potential (deprivation-weighted water consumption)	1.82E+14	2.63E+04	recalculated by Bjørn (2017)	Gerten et al. (2013)	Climate action, water and terrestrial life protection
Particulate matter	PM	Disease incidence	Impact on human health	5.16E+05	7.47E-05	based on the environmental burden of Vargas-Gonzalez et al. (2019)	WHO (2006)	Human health
Photochemical ozone formation, human health	POF	kg NMVOC eq	Tropospheric ozone concentration increase	4.07E+11	5.88E+01	recalculated by Bjørn (2017)	EEA (1998)	Human health
Human toxicity, cancer	HTOX_c	CTUh	Comparative Toxic Unit for humans	9.62E+05	1.39E-04	based on the environmental burden of Vargas-Gonzalez et al. (2019)	WHO (2006)	Human health
Human toxicity, non-cancer	HTOX_nc	CTUh	Comparative Toxic Unit for humans	4.10E+06	5.93E-04	based on the environmental burden of Vargas-Gonzalez et al. (2019)	WHO (2006)	Human health
Ecotoxicity, freshwater	ECOTOX	CTUe	Comparative Toxic Unit for ecosystems	1.31E+14	1.90E+04	Bjørn and Hauschild (2015)	EC (2011)	Climate action, water and terrestrial life protection
Ionising radiation, human health	IR	kBq U^235^ eq	Human exposure efficiency relative to U^235^	5.27E+14	7.62E+04	based on the environmental burden of Vargas-Gonzalez et al. (2019)	WHO (2006)	Human health
Resource use, fossils	FRD	MJ	Abiotic resource depletion – fossil fuels (ADP-fossil)	2.24E+14	3.24E+04	JRC calculation based on factor 2 concept	Bringezu (2015); Buczko et al. (2016)	Resource use
Resource use, mineral and metals	MRD	kg Sb eq	Abiotic resource depletion (ADP ultimate reserves)	2.19E+08	3.18E-02	JRC calculation based on factor 2 concept	Bringezu (2015); Buczko et al. (2016)	Resource use

a Global population in 2010: 6,916,183,482 people, as in Bjørn and Hauschild (2015).

b Indicator description according to the Environmental Footprint recommendations (EC, 2017).

Bjorn (2017) refined some LCIA-based PBs, updating Bjørn and Hauschild (2015). This refinement process employed a conversion factor detailed in Equation (1).

(1) $$Conversion\;factor=\;\sum_{i=0}^{n}\frac{CF_{ILCD,\;i}}{CF_{EF,\;i}}\;x\;GNR_{EF,\;i}$$

The conversion factor is the sum of the substance-specific ratios between the characterization factor (CF) in Bjørn and Hauschild (2015) (ILCD method, CF_ILCD_) and in this study (EF method, CF_EF_) weighted according to the contribution of each elementary flow (*i*) to the global normalisation reference of the EF method (Sala et al., 2017; Crenna et al., 2019) underpinning the specific impact category.

#### 2.2.1 Planetary boundaries related to human health impacts

The LCIA-based PBs related to human health (linked to SDG 3) impacts included the following EF impact categories: photochemical ozone formation, human toxicity (cancer and non-cancer effects), particulate matter, and ionising radiation. While photochemical ozone formation was based on Bjørn and Hauschild (2015), the other human-health related LCIA-based PBs were defined following Vargas-Gonzalez et al. (2019).

The LCIA-based PB for photochemical ozone formation was refined by Bjørn (2017), based on the previous calculations by Bjørn and Hauschild (2015). The conversion factor (Equation (1)) was calculated based on the elementary flows contributing to this impact category (e.g., non-methane volatile organic compounds (NMVOC)). Although considered in this paper regarding human health impacts, POF may be also considered as intermediate for ecosystems quality.

The other four LCIA-based PBs for human health, namely human toxicity (cancer and non-cancer effects), particulate matter, and ionising radiation, are based on the concept of “acceptable environmental burden” of disease (Vargas-Gonzalez et al., 2019). The acceptable environmental burden of disease is measured in DALYs (Disability-Adjusted Life Years) and sets the limits for tolerable perturbation to human health as the number of DALYs that is possible to attribute to environmental factors (Knol et al., 2009). Due to a lack of relevant policy-oriented data for defining a specific environmental limit for each of these four impact categories and to have a coherent approach across them, the same acceptable environmental burden was chosen by Vargas-Gonzalez et al. (2019) (Table 4). The burden was defined based on the concentration of PM_2.5_ estimated as tolerable for a healthy environment (i.e. 10 μg m^−3^ as recommended by WHO (2006)): 0.0016 DALYs.

**Table 4:** Details underpinning the planetary boundaries for human health, based on the work done by Vargas-Gonzalez et al. (2019).

EF Impact category	EF Unit	Acceptable environmental burden (DALY. person^−1^. yr^−1^)	Conversion factor (DALY. EF Unit^−1^)
Human toxicity, cancer	CTUh	1.60E-03	1.15E+01
Human toxicity, non-cancer	CTUh	1.60E-03	2.70E+00
Particulate matter	Disease incidence	1.60E-03	2.14E+01
Ionising radiation	kBq^235^U eq.	1.60E-03	2.10E-08

**Global population in 2010: 6,916,183,482 people, as in Bjørn and Hauschild (2015).

Based on the acceptable environmental burden, the LCIA-based PBs for these impact categories were then calculated by translating the value in DALYs into EF metrics, by using the conversion factors proposed by Vargas-Gonzalez et al. (2019) (Table 4). However, a different conversion factor was calculated for particulate matter, as the metrics in the EF method were not coherent with the ones used in Vargas-Gonzalez et al. (2019). The conversion factor for particulate matter was calculated as the ratio between the CF at endpoint and the CF at midpoint for the elementary flow PM_2.5_ to air, emitted at ground level (Fantke et al., 2016). Such choice was based on a precautionary principle, as this CF brings the highest impacts for particulate matter.

Air pollution in both urban and rural areas has been shown to cause significant health risk at global level, including premature death, especially due to the exposure to small particulate matter (PM_2.5_) (WHO, 2018). Due to the relevance of this impact category and the availability of additional data and sources (Vargas-Gonzalez et al., 2019; Ryberg, 2018), alternative calculations of the corresponding LCIA-based PB were explored to show the uncertainties behind this value. The estimates of the alternative LCIA-based PBs were still built on the definition of an “acceptable environmental burden”, or “tolerable damage level” as defined by Ryberg (2018), for particulate matter and respiratory inorganics effects. The estimated LCIA-based PBs were converted into EF metrics (namely disease incidence) by using differently calculated conversion factors.

The resulting LCIA-based PBs (Table 5) differed between them of up to six orders of magnitude, and the currently proposed LCIA-based PB for particulate matter falls in between the estimated alternative options (supplementary material SM2). The three proposed alternatives are hereafter detailed.

**Table 5:** Estimates of the LCIA-based planetary boundaries (PBs) for particulate matter in disease incidence referred to both the whole World population and per citizen.

	PB value [disease incidence]	PB value [disease incidence person^−1^]	Approach
Currently used	5.16E+05	7.47E-05	Based on the acceptable environmental burden defined by Vargas-Gonzalez et al. (2019) and the factor from Fantke et al. (2016)
Alternative 1 ^a^	3.12E+06	4.50E-04	Based on the tolerable damage level set by Ryberg (2018), and the factor from Fantke et al. (2016)
Alternative 2 ^b^	6.42E+01	9.28E-09	Based on PM_2.5_ environmental concentration (WHO , 2006), and the factor from Fantke et al. (2016);
Alternative 3 ^c^	8.67E+00	1.25E-09	Based on PM_2.5_ environmental concentration (WHO , 2006), and factor from EF 2017 method

Alternative 1 builds on the Tolerable midpoint Impact Score (TmidIS) (Ryberg, 2018), based on the environmental concentration of PM_2.5_ for a healthy environment (WHO, 2006) and the use of the TM5-FASST tool (European Commission - Joint Research Centre (EC-JRC), 2018). Then, the estimated emission was adapted to EF metrics by means of the conversion factor derived from Fantke et al. (2016), as used for the currently proposed LCIA-based PB. Equation (2) details the calculation of alternative 1 for the LCIA-based PB particulate matter, which includes the damage factor for respiratory effects of PM_2.5_ from Gronlund et al. (2015). The conversion factor DALY to Disease incidence was obtained through the ratio of the CFs endpoint and midpoint (CF endpoint/CF midpoint) from Fantke et al. (2016).

(2) $$PB_{\mathrm{PM},\;\mathrm{Alt}\;1}=TmidIS\;\left(\frac{kg\;PM_{2.5}\;eq}{capita*year}\right)x\;Global\;population\;\left(inhabitants\right)x\;DF_{resp.\;inorg}\left(\frac{DALY}{kg\;PM_{2.5}}\right)x\;\frac{1\;Disease\;incidence}{21.4\;DALY}$$

Alternative 2 and 3 represented worst-case-scenarios. Both based on the environmental concentration of PM_2.5_ for a healthy environment (C_WHO_) (WHO, 2006), it is assumed that every kilogram of PM_2.5_ emitted within the technosphere is inhaled by humans, thus causing damage to human health. Therefore, no fate was considered between emission to the atmosphere and human inhalation.

In alternative 2 (Equation (3)), the amount of PM_2.5_ emitted from natural sources (e_ns_) (18%, Vargas-Gonzalez et al., 2019) has been deducted from the WHO value to consider only the emissions from non-natural sources in the boundary estimation. For calculating the total emissions of PM_2.5_ at the global level, this concentration was multiplied by the average inhalation rate of a person (InR) (13 m^3^.person^−1^. day^−1^, as in USEtox (Rosenbaum et al., 2008)), the global population in 2010 (Bjørn and Hauschild, 2015) and the factor 365 days·year^−1^. The global emission of PM_2.5_ was converted into EF metrics (i.e. disease incidence) by means of the CF of PM_2.5_ emitted at ground level from Fantke et al. (2016).

$$PB_{\mathrm{PM},\;\mathrm{Alt}\;2}=\left(\left(1-e_{ns}\right)\;x\;C_{WHO}\;\left(\frac{\mu g\;PM_{2.5}}{m^{3}}\right)\right)x\;\;\frac{1\;kg}{10^{9}\;\mu g}x\;InR\;\left(\frac{m^{3}}{capita*day}\right)x\;\frac{365\;days}{1\;year}x$$

(3) $$Global\;population\;\left(inhabitants\right)x\;CF_{PM2.5,\;ground}\left(\frac{Disease\;incidence}{kg\;PM_{2.5}}\right)$$

Alternative 3 (Equation (4)) was calculated with the same rationale as alternative 2. However, instead of employing the CF of PM_2.5_ emitted at ground level (Fantke et al., 2016), the damage factor for respiratory effects inorganics (Gronlund et al., 2015) and the conversion factor DALY to Disease incidence as in alternative 1 were employed.

$$PB_{\mathrm{PM},\;\mathrm{Alt}\;3}=\left(\left(1-e_{ns}\right)\;x\;C_{WHO}\;\left(\frac{\mu g\;PM_{2.5}}{m^{3}}\right)\right)x\;\;\frac{1\;kg}{10^{9}\;\mu g}x\;InR\;\left(\frac{m^{3}}{capita*day}\right)x\;\frac{365\;days}{1\;year}x$$

(4) $$Global\;population\;\left(inhabitants\right)x\;DF_{resp.\;inorg}\left(\frac{DALY}{kg\;PM_{2.5}}\right)x\;\frac{1\;Disease\;incidence}{21.4\;DALY}$$

#### 2.2.2 Planetary boundaries for climate action, water and terrestrial life protection

The LCA-based PBs for climate change and ozone depletion (linked to climate action, SDG 13) as well as the ones for eutrophication -both marine and freshwater- and ecotoxicity (linked to water and aquatic life protection, SDG 14) were taken from Bjørn and Hauschild (2015). The metrics adopted in their study were already in line with the EF method.

Regarding climate change, no adaptation was required for the LCIA-based PB since the same impact assessment model (i.e. IPCC, 2013) was used in Bjørn and Hauschild (2015) and the EF method. The 2-degree Celsius climate threshold was used as in Bjørn and Hauschild (2015).

The ecological boundary on soil erosion employed in Bjørn and Hauschild (2015) was used to define the LCIA-based PB for land use. Since the metrics of this boundary (kg soil loss) differ from the ones in the EF method (Pt), the impact of land use was assessed using the soil erosion indicator in the LANCA model (Horn and Maier, 2018) as updated in De Laurentiis et al. (2019). Regarding global environmental impacts (section 2.1.4), the value for this category was also adapted to the new metrics. The LANCA model for soil erosion was applied to the global inventory (Crenna et al., 2019), resulting in a global normalisation factor for land use of 7.82·10^14^ kg soil loss.

The LCIA-based PBs for terrestrial eutrophication, acidification and water use were re-calculated, as suggested by Bjørn (2017), in order to adapt the value calculated by Bjørn and Hauschild (2015) to EF metrics following a conversion factor (Equation (1)). The following elementary flows were involved in the recalculation process for each category:

- terrestrial eutrophication: ammonia and nitrogen oxide;
- acidification: ammonia, nitrogen dioxide and sulphur dioxide;
- water use: freshwater.

The CFs of the EF method differed from the ones used by Bjørn and Hauschild (2015) in their estimates, i.e. the ILCD ones (EC-JRC, 2012), due to either an update or a replacement of the models behind the CF estimates. Particularly for the water use impact category, the EF method is based on novel impact assessment models and indicators (i.e. deprivation-weighted water consumption instead of water withdrawal). Therefore, differences lie in the rationale behind CF estimation, leading to the need of a conversion factor. Other ways of calculating a substance-generic conversion factor from substance-specific ratios are certainly possible and may result in quite different values.

#### 2.2.3 Planetary boundary for resource use

As the LCIA-based PBs for the “resource use” impact categories were not included in the available literature, they were calculated by applying the concept of Factor 2 to the global normalisation reference for resources (Sala et al., 2017; Crenna et al., 2019). In fact, according to Buczko et al. (2016) and Bringezu (2015), a reduction in material consumption by a factor 2 (namely 50%) at the global level is needed to achieve environmental sustainability. Contrary to the other impact categories, the principle applied for resources use is more normative than the boundary or carrying capacity approach.

#### 2.2.4 Zone of uncertainty of LCIA-based planetary boundaries

The resulting LCIA-based PBs refer to the limit of the safe operating space and, therefore, the lower limit of the zone of uncertainty defined by Rockström et al. (2009). Since a defined zone of uncertainty is not available for all the LCIA-based PBs, a fixed zone of uncertainty has been set as two times the PB, i.e. the area between the limit of the safe operating space and the double of the respective PB. Thus, the safe operating space is equal to the zone of uncertainty. Beyond the upper limit of the zone of uncertainty (>2 times the PB), there is the area at high risk. Within this area at high risk, the graphical representation of the results is limited to up to 3 times the PB. For those LCIA-based PBs with correspondence to the framework of Rockström and colleagues, a sensitivity analysis was performed on employing the same zone of uncertainty in the analysis (see section 4.2).

### 2.3 Ethical dimension: allocating the planetary boundaries to the EU

Different allocation methods can be applied when assessing the PBs to lower scales than the entire globe. As investigated by Ryberg et al. (2018b), the selection of the allocation method was the main uncertainty source when employing the PB-LCIA method. Häyhä et al. (2016) reported different allocation pathways, including different ethical principles (i.e. equality, sovereignty, right to development, responsibility, capacity and voluntarism). Due to the uncertainty in allocation procedures to downscale PBs to lower geographical scales, results were analyzed using two different approaches. First, the results were contrasted to the PBs at the global scale in order to observe the current situation at the global level and the role of EU (Section 3.1). Second, the equality allocation method, where all the population have equal rights to the environment and therefore the PB is distributed equally, was selected in order to assess the environmental impacts against the LCIA-based PBs at the EU scale (Section 3.2). This allocation procedure was selected due to the high presence in PB studies (e.g. Dao et al., 2018; Hoff et al., 2014).

## 3 Results

The results of the LCA-based indicators were compared with both global normalisation references and LCIA-based PBs adapted to EF metrics, apart from the land use category (employing the metrics kg soil loss). Results per impact category are extensively reported in the supplementary material (SM3).

### 3.1 The relevance of the environmental impacts of EU consumption at the global level

**Fig. 3:**
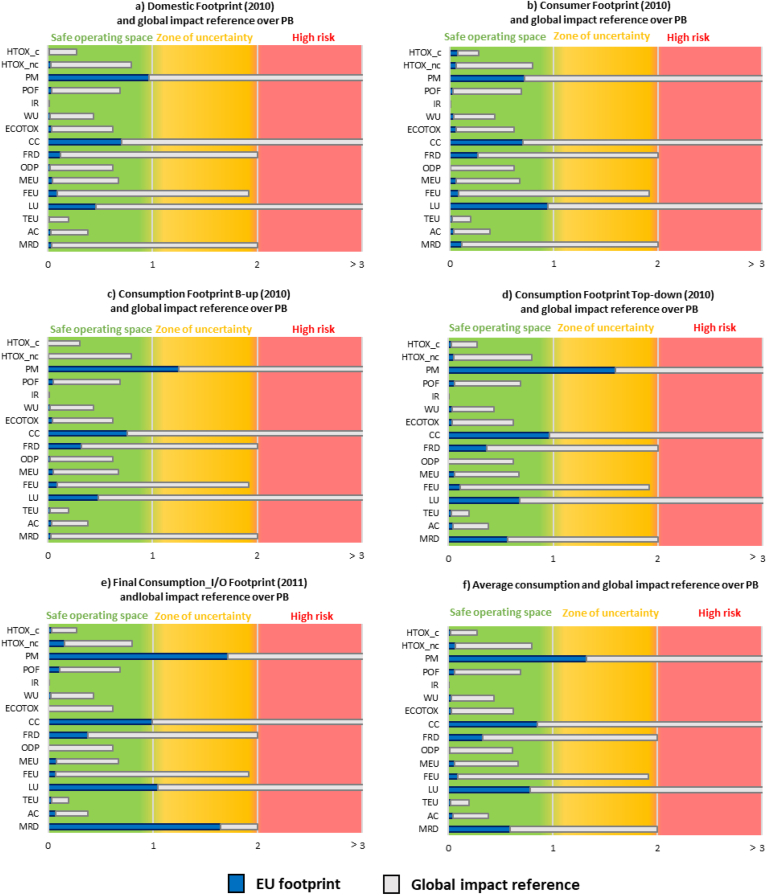
Life cycle indicators' results, as total impacts, compared to global impacts and planetary boundaries (PBs). The colour code of the background reflects the status of the planetary boundary for each impact category: green = below the PB; orange = within the zone of uncertainty of the PB; red = in a high risk area. Acronyms of the EF impact categories refer to the ones presented in Table 3. The extent to which every impact category overcomes the related boundary is reported in brackets. (For interpretation of the references to colour in this figure legend, the reader is referred to the Web version of this article.)

The overall environmental impact of EU consumption and the global normalisation references (Sala et al., 2017; Crenna et al., 2019) were assessed against the LCIA-based PBs for the EF impact categories (Fig. 3). The global references (in grey) surpassed the safe operating space by sixty times for land use (LU) and eight times for particulate matter (PM) and climate change (CC). The values of the global impacts in other three indicators (fossil resource depletion, freshwater eutrophication, and mineral resources depletion) were found within the zone of uncertainty. For the rest of the indicators, global environmental impacts remained within the safe operating space, being ionising radiation the one with the lowest value.

Analysing the contribution of the impacts of EU consumption within the global reference unveiled a different EU contribution depending on the impact category and to the adopted LCA-based indicator. When comparing the impacts generated at EU level against the global impacts, the EU contribution spanned from 1.3% of the land use impact to 45% of ionising radiation impact, on average (Fig. 3f). This resulted from differences in production and consumption patterns between the EU and the rest of the world, e.g. the relevant role of the EU in nuclear energy at the global scale justifies the high contribution in this impact category. At the global level, trade flows can affect the environmental impacts of consumption as the environmental profile of products can vary among countries, e.g. imports to the EU from emerging countries with weak environmental policies may lead to products with higher environmental impact intensity. However, the effect of imports in the overall Consumption Footprint depends on the consumption intensity of these products by EU citizens. In this sense, shifting consumption to products from a diverse origin may affect the resulting environmental impact of consumption.

The low share of ozone depletion was given by the bottom-up modelling approaches, as the top-down models do not include this impact category due to lack of MRIO data. The considerable impact of ionising radiation at EU level is driven by the emissions of carbon-14, which represents the most important flow in terms of impact both at European and at global scales for this category (Crenna et al., 2019). In fact, most of carbon-14 emissions registered at global level stems from nuclear power plants installed only in Europe and operating for electricity production, contributing to near 60% of global carbon-14 emissions (UNSCEAR, 2017).

Focusing on the role of the environmental impacts of EU consumption among the global environmental impacts and their relation to the quantified LCIA-based PBs (Fig. 3), the analysis revealed that for some impact categories, the environmental burdens generated by EU citizens already transgressed the global PBs, i.e. particulate matter (Consumption Footprint bottom-up, Consumption Footprint top-down and Final Consumption I/O Footprint) and mineral resources use (Final Consumption I/O Footprint). Along the same lines, the impact of EU consumption for climate change represented more than 90% of the safe operating space globally available, thus, leading to a very critical situation, i.e. limited space left available to the rest of the world.

These outputs were related to two main aspects. Firstly, the large global impacts in some LCIA-based PBs (i.e. particulate matter, land use and climate change) suggested that structural transformations might be required to improve the performance not only at the EU but also at the global level. Secondly, data limitations of the Exiobase database regarding the granularity of mineral and metals flows have contributed to higher uncertainties in this impact category (i.e. resource use, mineral and metals), which could have led to overestimated impacts (Sala et al., 2019a).

On the SDGs, the environmental impacts of EU consumption showed the worst performance in SDG 3 (human health), SDG 15 (terrestrial ecosystems) and SDG 13 (climate action), where LCIA-based PBs were transgressed. Considering the global environmental impacts, also SDG 14 (water ecosystems) showed environmental impacts within the zone of uncertainty (e.g. freshwater eutrophication) of the LCIA-based PBs. On the contrary, environmental impacts related to SDG 6 showed the best results with impacts within the safe operating space (Fig. 3).

### 3.2 Are the environmental impacts of EU consumption beyond the safe operating space?

Towards assessing the environmental impacts of EU consumption against the LCIA-based PBs, a downscale procedure to define the allocation of the global PBs to a lower geographical scale was required. Following an equality principle of sharing the PBs (Häyhä et al., 2016), Fig. 4 displays the environmental impacts of an average EU citizen and an average global citizen against the LCIA-based PBs per capita.

**Fig. 4:**
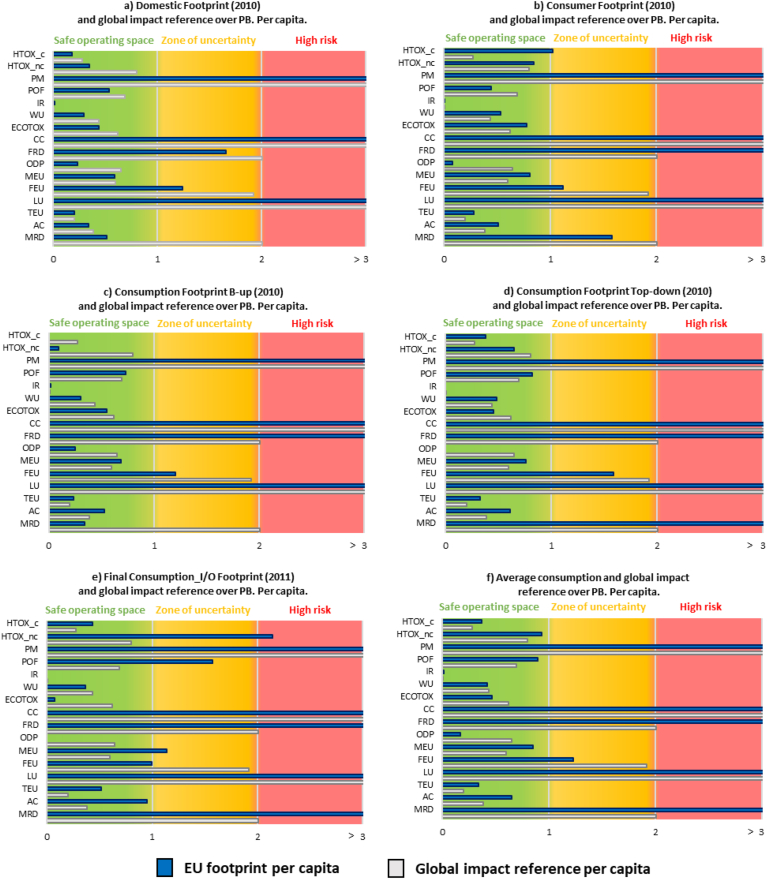
Life cycle indicators' results, as impacts per capita, compared to global impacts and planetary boundaries (PBs). Colour code is explained in the figure above. Acronyms for the EF impact categories refer to the ones presented in Table 3. The extent to which every impact category overcomes the related boundary is reported in brackets. (For interpretation of the references to colour in this figure legend, the reader is referred to the Web version of this article.)

EU citizens do not appear to be “living within the limits of our planet”, as also observed by Häyhä et al. (2016). In fact, a significant transgression of the LCIA-based PBs per capita was observed with all adopted modelling approaches for assessing the environmental impacts of EU consumption. The LCIA-based PBs per capita were transgressed in many impact categories, being exceeded from four times (e.g. fossil resource use in the bottom-up approaches) to more than twenty times (e.g. particulate matter in the top-down approaches).

The environmental impacts of EU consumption were associated to the high consumption intensity of an average EU citizen. The results of an average EU citizen were generally higher than the impacts of an average world citizen for many impact categories, apart from land use, freshwater eutrophication, ecotoxicity, fossil resources depletion and ozone depletion potential. These results can be linked to many factors, as for instance the role of developed economies (e.g. high consumption oriented behavior, such as high fossil fuel consumption), the role of developing economies (e.g. deforestation in rainforest areas) and the different efficacy of environmental policy among countries.

Furthermore, different principles other than equal per capita exists for allocating the PBs to geographical territories (e.g., in this case the EU territory), based on normative choices and socio-economic indicators, which may influence the outcome (Häyhä et al., 2016; Ryberg, 2018). The use of PBs in the policy-making process should consider changes in scale and downscaling pathways, which may lead to considerably different results and interpretations.

## 4 Discussion

The analysis of the results unveiled four main aspects to be further discussed: the relevance of PBs in assessing the environmental impacts of EU consumption and the policy implications, the uncertainties of the assessment, the role of PBs in LCA and the possible ways to integrate PBs into LCA.

### 4.1 Results of the study and policy implications

The results presented in this study modelled the EU consumption as a whole, without differentiating among areas of consumption. Towards addressing policy needs, disaggregating the environmental contributions by consumption area is essential. Among the assessed indicators, the LCA-based indicator Consumer Footprint allows for assessing the contribution of five different areas of consumption (i.e. food, mobility, housing, household goods and appliances) (Sala et al., 2019a). Among these areas of consumption, food was one of the predominant contributor to the European impacts within the Consumer Footprint (Sala et al., 2019a) toghether with housing and mobility. In fact, food gained major interest in the current literature asa key driver of environmental impacts (e.g. Springmann et al., 2018; Chandrakumar et al., 2019). At global level, the food system represented the main driver of climate change, land use, and impacts on terrestrial and aquatic ecosystems, e.g. due to eutrophication (Springmann et al., 2018). This was also reflected at European level, where the impacts of food consumption spanned from 33% of the total Consumer Footprint for climate change, to 74% for eutrophication (both marine and terrestrial) (see SM4) (Sala et al., 2019a). Based on these results, policy actions concerning the PBs might consider the area of consumption of food as a priority due to the high relevance among the impacts of EU consumption.

PBs showed a high potential to support policy-making as absolute sustainability thresholds for defining policy targets. According to the results, policy actions regarding land use, climate change and particulate matter might be prioritized as all the LCIA-based indicators assessed situated those environmental impacts in the high-risk area.

Mainstreaming the use of PBs in policy-making might overcome certain barriers. In fact, a proper allocation to territorial boundaries might be defined for the operationalisation of the global PBs at lower scales. This study employed the most widely used ethical principle for allocating PBs to lower scales: equity. However, other ethical principles can be employed (e.g. sovereignty, right to development) (Häyhä et al., 2016) which can vary the resulting PBs for a certain area. As for other environmental issues, such as carbon emissions quotas, different allocating processes have been discussed in the literature (Raupach et al., 2014). The employment of PBs in policy might therefore foreseen an agreement on allocation procedures for their implementation at regional and national scales.

PBs-based policy targets towards identifying the sustainability gap between current trends and desirable outputs would contribute to the employment of science-based targets in policy. In fact, the Science-Based Targets Initiative has promoted science-based targets for greenhouse gas emission reduction targets by using data from climate science towards ensuring effective actions (Science based targets, 2019). The PBs framework would therefore contribute to science-based targets by covering further Earth's processes that should be considered in forthcoming environmental policies.

For example, focusing on the Consumer Footprint, food consumption represents 35% of the total carbon footprint of an average EU citizen, being equal to approximately 3.4 tonnes CO_2_eq per year. However, to meet the PB on climate change, a limit of around 1 ton of CO_2_eq per citizen per year (985 kg) has been set. Distributing equally the effort of impact reduction among sectors of consumption would mean a target of CO_2_ eq emission per citizen for food equal to 350 kg, meaning a 90% reduction compared to the current situation, basically a factor 10. This result is converging with conceptual targets set on sustainability, such those of the so-called “Factor 10” (see e.g. Schmidt-Bleek, 2008), substantiating this quantitatively. This target based on absolute sustainability, rather than on relative one, highlights the extent of the effort needed by production and consumption systems to remain within PBs.

### 4.2 Uncertainties

Noticeably, uncertainties exist when coming to quantify the production and consumption systems and their sustainability. In the LCA domain, uncertainty can be identified at three main level, namely: i) inventory; ii) LCIA models, and ii) LCIA-based PBs definition.

Concerning the inventory of pressures, the uncertainties were linked with the collection and modelling at both EU and global scales. In fact, data were often incomplete from e.g. the temporal or geographical point of view, or based on estimations and prediction models, thus limiting the reliability and robustness of the final results (as shown in Benini and Sala, 2016). For instance, considering the global impact reference for marine eutrophication (1.35·10^+^^11^ kg N eq.) (Crenna et al., 2019), this value is not properly in line with the current global picture for biogeochemical flows. According to the PBs framework by Steffen et al. (2015), the global nitrogen limit has been already outreached, being far beyond the zone of uncertainty; while the global impacts were within the safe operating space of the LCIA-based PB. This may be due to a poor availability of data underpinning the calculation of global impacts (Crenna et al., 2019).

For what concerns the impact assessment methodology, the underpinning LCIA models of the EF method are characterised by uncertainties, which may influence to different extents the robustness of the 16 impact categories (EC, 2017). Although uncertainty has not been quantified for the LCIA-based PBs, the robustness level of the different impact categories qualitatively indicated the potential uncertainty of each indicator. According to the recommendations of the EF method, those impact categories with lower robustness are human toxicity (both cancer and non-cancer), ecotoxicity (freshwater), land use, water use and resource use (both fossils and minerals and metals) (EC, 2017).

Finally, concerning the LCIA-based PBs, uncertainties arose because PBs are based on limits associated to ecological processes, which by nature are difficult to quantify and attribute to the specific LCIA features. This is particularly related to the difficulties in scaling up local environmental pressures to the global level of PBs (Bjørn and Hauschild, 2015; Springmann et al., 2018). A clear example of uncertainty at this level is observed for particulate matter, for which the currently proposed LCIA-based PB falls in between the estimated alternative options. The first alternative, based on Ryberg (2018), is not far from the currently proposed boundary, although being one order of magnitude higher. This option was calculated with a different modelling approach to derive the PM_2.5_ emissions from the initial environmental concentration, thus leading to different results. In fact, Ryberg's modelling has a simplified spatial resolution, namely the predicting model does not consider the differentiation between emissions in a urban context versus those in a rural and remote areas. On the other hand, the alternatives 2 and 3 values appear to be very low, with all the consumption-modelling approaches surpassing them of more than thousands times both when considering the total and per capita impacts (see SM5). These ratios seem out of scale compared to the LCIA-based PBs for the other indicators, such as climate change for which all the consumption-modelling approach are generally close to the LCIA-based PBs when considering the total impacts and exceed the planet limit at least eight times when considering the impacts per capita. According to this explorative exercise, the different alternatives for the estimation of the LCIA-based PB for particulate matter enlarged the range of results rather than suggesting a specific pathway, thereby increasing the uncertainty behind this value.

Another source of uncertainty relies on the calculation of the LCIA-based PBs, the use of the concept boundary and its relation to the framework developed by Rockström et al. (2009). While this study presents and evaluates the PBs following the concepts of Rockström et al. (2009), some impact categories are based on the carrying capacities established by Bjørn and Hauschild (2015), which also employ sources beyond Rockström's framework (SM6). Furthermore, when calculating the carrying capacities (Bjørn and Hauschild, 2015) in relation to the same environmental aspect as Rockström (e.g. climate change: limit of temperature increase), the environmental limits were not always equally set. This can only be detailed for climate change, ozone depletion and water use (SM6). Some LCIA-based PBs considered a lower limit than Rockström (e.g. ozone depletion, water use), while some zones of uncertainty were larger (climate change) or smaller (water use). As sensitivity, the boundary and zone of uncertainty of these impact categories were adjusted to the values defined by Rockström et al. (2009) and compared to the ones used in this study for the Consumer Footprint and the Consumption Footprint top-down (SM7). The recalculation does not affect the global results nor the impacts per capita, as climate change still shows a high risk beyond the zone of uncertainty and both ozone layer depletion and water use remain within the safe operating space.

Besides, one may question, as already stated by Hoff et al. (2014), how appropriate could be to assess the contribution of European consumption to the transgression of PBs beyond truly global impacts such as climate change or ozone depletion. For all the other boundaries, the spatial variability of pressures and impacts is such that it is questionable how representative the result of the comparison can be.

### 4.3 Planetary boundaries and LCA

The current set of defined PBs is not matching with all the usually adopted LCIA impact categories. First, some PBs are basically pressure-based (e.g. those related to biogeochemical fixation) or are adopting as indicator an endpoint indicator in LCA terms (e.g. the biodiversity) (Chandrakumar and McLaren, 2018; Dong and Hauschild, 2017). Secondly, LCIA frameworks are based on the identification of a cause-effect chain that is connecting pressure to potential impacts by means of midpoint and endpoint indicators, aiming at making explicitly causalities between the different steps (Ryberg et al., 2018b). Despite some boundaries are interconnected in the PBs framework, they are still presented as separated items (Bjørn and Hauschild, 2015). For example, biodiversity is affected by the transgression of the other PBs (e.g. climate change, land use, biogeochemical flows perturbation).

Besides, the capability of the PBs to capture the breadth of elementary flows available in commonly used life cycle inventories (LCIs) is very limited. For example, there is a gap between the control variable ppm of CO_2_ (PB climate change) with the at least 40 different greenhouse gases listed in LCIs. Regarding land use flows, the control variables in the PBs framework focus on the forest system, while more than 20 different elementary flows are available in LCIs. Additionally, the majority of elementary flows in LCIs are related either to chemicals, for which a PB on novel entities is not defined yet, and for resources (such as mineral and metals), not included in the PBs framework. Therefore, using LCIA-based PBs for assessing the absolute sustainability of systems represents also an opportunity towards detailing aspects of the Earth processes considered in the PBs, enabling a quantitative assessment. Particularly, this advantage is crucial for PBs without a threshold defined yet, e.g. novel entities can be assessed through human toxicity or ecotoxicity categories.

In fact, the different scopes of LCA and the PBs framework are highlighted. LCA covers three areas of protection including human health, ecosystems quality and natural resources. While some environmental pressures can result in impacts on human health (e.g., novel entities and human toxicity), some impact categories remain beyond the coverage of the PBs (e.g., resources depletion). Notwithstanding that the attempts to cover those LCA impact categories employ the rationale of having a “safe operating space” boundary, these values are beyond the scope of the PBs framework.

Finally, aspects related to the temporal and spatial dimension of the impacts modelled in the LCA framework and the control variables in the PB framework remain challenging to bridge (Ryberg et al., 2016). For example, in LCA the impacts occurring along the life cycle of a product are assessed as they are happening simultaneously, whereas there could be a time lag (e.g. the end of life of a building after 100 years). PBs are related to a specific timeframe and are not looking at multiple temporal scales. On the spatial scale, regionalisation of impacts is more and more central in LCIA, although control variables in the PB framework are mainly assessed as global boundaries.

Current discussions in the literature on the application of absolute metrics to LCA is looking at how to improve the application of PBs in LCA and how to fill the gap in relation to current boundaries missing in the framework. In any case, a sensitivity between different approaches to define PBs is a possible outlook to reduce the huge uncertainty associated with their application.

### 4.4 Using the planetary boundaries in LCA

To date, PBs have mainly been used in LCA as normalisation reference for measuring absolute sustainability of products or services (Bjørn and Hauschild, 2015; Bjørn et al., 2015). However, other ways can be explored in which the concept of PBs may be integrated into LCA, such as being adopted as a basis for a specific impact assessment method (e.g. Fang et al., 2015; Chandrakumar and McLaren, 2018; Ryberg et al., 2018a,b). This would require adapting characterisation models to account for the specificities and metrics of the control variables of PBs (Ryberg et al., 2018a,b). This is linked to the already identified problem of developing and operationalizing an absolute impact assessment methodology within LCA that enables the application of the PB framework for decision support in policy planning (Sala et al., 2013b; Clift et al., 2017). The PB-LCIA (Ryberg et al., 2018a) includes 85 CFs derived from existing levels of environmental pressures for 16 impact categories. This method is still under development, due to several challenges related to its operationalisation, from adapting LCIs to the best operational definition of the safe operating space for sustainability assessment.

Alternatively, PBs may be used as a basis for setting weighting factors (Goossens et al., 2018; Tuomisto et al., 2012; Vargas-Gonzalez et al., 2019). In fact, distance-to-target (DTT) approaches based on carrying capacity are methods for developing weighting factors. Through the DTT approach, the magnitude of impacts compared to the existing PBs has been scored by experts to derive weights on midpoint level within the EF context (Sala et al., 2018). However, this current set has not been agreed within the LCA community and, thus, is still not mature enough for being recommended.

## 5 Conclusion and outlook

The environmental impacts of EU consumption in 2010 have been put in perspective with the PBs framework to assess the absolute environmental sustainability of Europe. In fact, measuring to which extent Europe is within the Earth's carrying capacity is needed in order to move towards more responsible consumption and production (SDG 12) and ensure “living well within the limits of our Planet” (EC, 2013).

The environmental impacts of EU consumption surpassed the LCIA-based PBs for several categories, as climate change, land use and particulate matter were found in the high-risk area of the corresponding LCIA-based PB. When considering PBs in policy-making, these results should highlight the necessity for policy actions addressing impacts in these categories. Moreover, studies, like the one conducted here, are fundamental to support the assessment of future scenarios that may help investigate feasible and optimal consumption patterns for staying within the PBs.

PBs can be adopted in the policy-making process. Indeed, their knowledge would improve environmental policy relevance by employing science-based targets, which allows quantifying the sustainability gap between current impacts and related carrying capacity-based limits. Nevertheless, criticalities may raise for the difficulties and the consequent uncertainties in determining PBs. Indeed, they are based on complex ecological mechanisms, which are by nature difficult to evaluate and, then, translated into the specific metrics used in LCA.

Regarding the selection of the impact reference values to be compared with the PBs, every approach to the estimation of level of pressure and impacts to the environment may have pro's and con's. Indeed, it is already relevant to observe certain convergence of the results, when comparing the EU consumption measured through the different LCA-based indicators with the available LCIA-based PBs. Further research is needed to overcome the uncertainties and the limitations at the different levels, and to improve the completeness of results. This may entail improving the coverage of missing impact categories and the assessment of the nexus and interplay between impact categories as well as the feedback into ecosystems due to the impacts in a specific category/boundary (Karabulut et al., 2018).

There is a vivid debate in the LCA community on the need of addressing aspects related to absolute sustainability, namely moving the environmental assessment of products from a relative perspective towards the integration of concepts related to planetary limits (Hauschild, 2015). In fact, the PBs framework could contribute to define a common platform for decision-makers, supporting communication and strategic policy planning toward a more concrete environmental sustainability assessment. Coupling PBs with LCA show a high potential to support policy-making, especially for what concern the definition of absolute sustainability thresholds and related policy targets.

## CRediT authorship contribution statement

**Serenella Sala:** Conceptualization, Methodology, Validation, Writing - original draft, Writing - review & editing. **Eleonora Crenna:** Formal analysis, Investigation, Visualization. **Michela Secchi:** Data curation, Formal analysis. **Esther Sanyé-Mengual:** Methodology, Writing - review & editing.

## Declaration of competing interest

None.
