# Corrigendum to “Environmental sustainability of European production and consumption assessed against planetary boundaries”

**DOI:** 10.1016/j.jenvman.2020.111904

**Published:** 2021-03-01

**Authors:** Serenella Sala, Eleonora Crenna, Michela Secchi, Esther Sanyé-Mengual

**Affiliations:** European Commission, Joint Research Centre (JRC), Via E. Fermi 2749, 21027, Ispra, Italy

**Keywords:** Consumption patterns, Impact assessment, Life cycle assessment based indicator, Sustainable development goals, Absolute sustainability, Carrying capacity

## Abstract

• Planetary Boundaries help quantify the environmental sustainability of consumption.
• We developed LCIA-based planetary boundaries for evaluating the EU consumption.
• EU consumption occupies a high share of the safe operating space globally available.
• Planetary boundaries are fundamental to support policy making towards sustainability.
• LCA-based planetary boundaries show intrinsic uncertainties.

The supplementary data file of the paper has been updated, with the following modifications:

- SM1: The value of the global normalization factor (NF) for land use is 7.82E+14.
- SM3: The values for the impact category Land use have been corrected for the total results (row 20) and per person results (row 39).
- SM4: The values for the impact category Land use have been corrected for the total results (row 20) and per person results (row 39).

The authors would like to apologise for any inconvenience caused.

## Declaration of competing interest

The authors declare that they have no known competing financial interests or personal relationships that could have appeared to influence the work reported in this paper.

